# Localized Corrosion Resistance on Additively Manufactured Ti Alloys by Means of Electrochemical Critical Localized Corrosion Potential in Biomedical Solution Environments

**DOI:** 10.3390/ma14237481

**Published:** 2021-12-06

**Authors:** Dong-Il Seo, Jae-Bong Lee

**Affiliations:** School of Advanced Materials Engineering, Kookmin University, 77 Jeongneung-ro, Seongbuk-gu, Seoul 02707, Korea; sdi1105@naver.com

**Keywords:** additive manufacturing, titanium alloy, E-CLCP, E-CLCT, repassivation

## Abstract

This study proposes a new method, electrochemical critical localized corrosion potential (E-CLCP), in order to evaluate localized corrosion resistance of biomedical additive manufacturing (AM) titanium (Ti) alloys. The procedures for determining E-CLCP are completely different from that of the electrochemical critically localized corrosion temperature (E-CLCT) method (ISO 22910:2020). However, its application should be limited to pH and temperature of the human body because of the temperature scan. E-CLCP displays the localized corrosion resistance of AM Ti alloys based on the human body’s repassivation kinetics, whereas E-CLCT displays the localized corrosion resistance of the alloys based on passive film breakdown in much harsher corrosive environments.

## 1. Introduction

Titanium (Ti) alloys with high strength-to-weight ratio and good corrosion resistance are used in marine, aerospace, and biomedical industries [1,2,3,4]. Due to their excellent biocompatibility, Ti alloys are the most promising biomedical materials in dental, acetabular, and other biomedical implant fields [5,6,7]. Conventionally, subtractive manufacturing (SM) methods are used to fabricate wrought or casted Ti alloys. However, new additive manufacturing (AM) methods (i.e., 3D printing) have replaced SM methods as they are more efficient and have reduced manufacturing costs [8]. The buy-to-fly ratio of AM to SM Ti alloys is approximately 20:1 [9]. From a mechanical view point, both the ductility and strength of AM Ti alloys are comparable with or higher than SM Ti alloys [10,11,12]. However, the resistance to corrosion of AM Ti alloys remains unknown and controversial. In addition, the localized corrosion resistance and repassivation characteristics of AM Ti alloys are vague and require further research. Considered distinct from SM Ti alloys, AM Ti alloys have anisotropic properties: The orientation, porosities, or martensite phases result from rapid cooling during stacking processes [13]. Acicular martensite α′ phases generated by rapid cooling during AM processes abruptly reduces the resistance to localized corrosion of AM Ti–6Al–4V alloys [14,15]. Although some studies on the corrosion resistance of bio-SM Ti alloys exist, only a few have been conducted on the resistance to corrosion of bio-AM Ti alloys [16,17,18,19]. Venugopalan and Gaydon [16] examined corrosion behaviors between SM Ti alloys and stainless steels in deaerated neutral Hank’s balanced salt solution at 37 °C via cyclic potentiodynamic polarization experiments. In comparison to that of 316 L (and multiple other stainless steels), the corrosion potential of Ti alloys is typically more noble. In addition, the corrosion rates of Ti alloys are lower, representing that a true potential-independent passive region is typically apparent in their polarization curve. Gurappa [17] reported the characteristics of Ti oxides and the corrosion resistance of SM Ti alloys in deaerated HBSS by measuring open-circuit potentials (OCP) and performing cyclic polarization tests. A thick TiO_2_ film (containing a low concentration of oxygen vacancies) represents a very slow mass transport rate across the film. Hanawa [18] studied the reconstruction and regeneration of various surface oxides on SM Ti alloys in biological environments. The results demonstrated that the repassivation rate was not affected by the variation of pH of the solution, the dissolved oxygen, or the proteins. Sharma et al. [19] investigated the uniform corrosion resistance of SM and AM Ti–6Al–4V alloys in various aqueous solutions (including simulated body fluid) by performing potentiodynamic polarization and electrochemical impedance spectroscopy (EIS). The results indicated that strict precautionary measures (e.g., modification of microstructure, scanning strategy, or barrier layer formation) must be taken to avoid the more severe corrosion damage to AM Ti–6Al–4V alloys that occurs in neutral or vigorously corrosive media. Morris et al. [20] studied the relation between corrosion and porosity associated with the improved biocompatibility and osseointegration of 3D-printed porous Ti in biomedical applications. They found that corrosion increases as porosity increases, maintaining the need for the existence of optimum porosity for biocompatibility. Seo and Lee [21,22,23,24,25,26] studied uniform and localized corrosion of AM Ti–6Al–4V alloys by conducting various electrochemical tests such as potentiodynamic polarization, EIS, electrochemical critical pitting temperature [27,28], electrochemical critical localized corrosion temperature (E-CLCT; ISO 22910:2020) [29], and electrochemical critical localized corrosion potential (E-CLCP; ISO/CD 4631:2021) [30]. The results indicated that the reduction in localized corrosion resistance of AM Ti alloys resulted from the formation of martensite α′ phases attributed to the rapid cooling that occurred during the AM fabrication processes. The results also demonstrated that the localized corrosion of AM Ti–6Al–4V alloys was completely different from that of SM Ti–6Al–4V alloys in terms of the shape and mechanism of localized corrosion. The AM alloys corrode layer by layer, whereas SM alloys corrode beneath the crevice formerly in the crevice assembly [31]. Based upon their experimental results and findings, the E-CLCT method was proposed: a new testing method for evaluating the existence of localized corrosion in AM Ti alloys [25,29]. They also proposed another new method, E-CLCP, for the evaluation of localized corrosion resistance in biomedical AM Ti alloys [30]. The E-CLCT method is the electrochemical testing method utilized to measure the critical temperature that breaks down the passive film during a temperature scan at low temperature in highly concentrated chloride (25 wt% NaCl) aqueous solutions. However, the use of the E-CLCT method to measure the resistance to localized corrosion of AM Ti alloys in artificial physiological fluids at 37 °C is somewhat questionable [32]. Thus, the new E-CLCP method evaluates the localized corrosion resistance of AM biomedical Ti alloys in simulated biomedical environments. International standards (e.g., ISO 10271 and ISO 16429) exist for testing the corrosion resistance of metallic biomaterials in human body environments [33,34]. Corrosion test methods have been demonstrated under ISO 10271 [33] (such as potentiostatic, potentiodynamic, and static immersion tests) in in vitro oral cavity models. Corrosion potential, break down potential, and chemical elemental analyses are used for biometals. However, this standard is not sufficient for evaluating the localized corrosion resistance of AM Ti alloys as they are quite different from conventional Ti alloys. ISO 16429 [34] specifies the measurement for the OCP of metallic implantable materials over extended periods in aqueous NaCl solutions. This measurement method is also simple enough to describe the localized corrosion behaviors of AM biomedical Ti alloys. Tsujikawa and Hisamatsu [35] evolved a step-wise method that depends on growing crevice corrosions and then gradually decreases the potential to clearly capture repassivation by evaluating the critical crevice potential (CCP). This method was complied with an American Society for Testing and Materials (ASTM) standard (G192) [36] and amalgamates the potentiodynamic, galvanostatic, and potentiostatic steps to allow for more control in the development and repassivation of localized corrosion. Even though the ASTM G192 test serves as an alternative method, this test is inappropriate for biomedical Ti alloys such as AM Ti–6Al–4V because the layer-by-layer corrosion instead of crevice occurs on the surface of the AM Ti alloys, as is distinct from crevice corrosions of stainless steels or nickel alloys [25]. Therefore, this study proposes a new method (ISO/CD 4631:2021) [30] for AM biomedical Ti alloys. This paper introduces an E-CLCP method based on the repassivation potential. The E-CLCP method proposes a new criterion for the evaluation of resistance relative to localized corrosion of biomedical AM Ti alloys in human body environments. This new method combines potentiodynamic, galvanostatic, and potentiostatic steps to allow for more control in the development and repassivation of localized corrosion. E-CLCP is regarded as the repassivation potential of AM Ti alloy samples without a hole and crevice former, measured in artificial physiological fluids. E-CLCP values are obtained as more accurate and reproducible than those measured by cyclic potentiodynamic polarization (CPP) [37,38,39,40]. E-CLCP [30] can be defined as the highest potential at which repassivation occurs, which is indicative of the resistance to localized corrosion in AM Ti alloy specimens. The higher the potential, the greater the resistance against localized corrosion. 

In this study, the validity of the new E-CLCP (ISO/CD 4631:2021) test method is confirmed and compared with the recently acknowledged E-CLCT test method (ISO 22910:2020) to determine whether it can evaluate the resistance to localized corrosion of AM biomedical Ti alloys. AM Ti–6Al–4V alloy samples heat treated at various temperatures are chosen in order to compare their values of E-CLCP with those of E-CLCT in terms of localized corrosion resistances. 

The mechanisms of resistance to localized corrosion on the as-received and heat-treated AM Ti–6Al–4V alloys are also investigated under E-CLCT and E-CLCP environments in order to clarify the differences in their behaviors under localized corrosion and repassivation. 

## 2. Materials and Experimental Methods

### 2.1. Sample and Solution Preparation

This study used as-received and heat-treated AM Ti–6Al–4V alloys as specimens. In addition to AM Ti–6Al–4V alloys, AM Ti–6Al–7Nb, AM CP Ti, and AM Ni 718 alloys were chosen for localized corrosion resistance comparison. The weight composition of the powder percentage of all test samples is shown in Table 1. All samples were cut and shaped into dimensions of 22 × 22 × 2 mm (thickness). The samples were fabricated by using a directed energy deposition process (InssTek, MX-4, Daejeon, Korea) with a laser power of 460 W at a scan speed of 0.85 m/min in an argon (Ar) gas atmosphere. AM alloys were stacked by means of concurrent injection with a laser. Two types of heat treatments were applied to the AM Ti–6Al–4V alloys. The first was furnace cooling after heating in a furnace for 2 h at temperatures of 650 °C, 750 °C, 850 °C, and 1000 °C, respectively [41]. First, Ar gas was used to purge a furnace of oxygen to minimize sample surface oxidation. The samples were then heated at a rate of 5 °C/min from room temperature to 650 °C (650 post heat-treated (HT)), 750 °C (750HT), 850 °C (850HT), or 1000 °C (1000HT). After the temperature was maintained for 2 h, each sample was furnace cooled to room temperature. The other type of heat treatment was water-quenched for rapid cooling following furnace heating to either 650 °C (650 water quenched (WQ)) or 750 °C (750 WQ) for 60 s [41]. All fabricated samples were wet-ground with 600-grit SiC paper, cleaned with double-distilled water, and air dried. Deaerated 5.7 M (25 wt%) NaCl aqueous solution for the E-CLCT tests [25,29] and deaerated Ringer solution (8.69 g/L NaCl, 0.30 g/L KCl, 0.48 g/L CaCl_2_) [32,42] were prepared at 37 °C, and aqueous 0.6 M (3.5 wt%) NaCl solution was prepared at 50 °C for the E-CLCP tests. EIS, the Mott–Schottky analysis, and abrading tests were performed in the deaerated Ringer solution.

### 2.2. Microstructural Characterization

In order to characterize microstructures, samples were etched at room temperature with a solution composed of 10 mL HNO_3_, 5 mL HF, and 85 mL H_2_O. An optical microscope (OM) (Nikon Eclipse LV100, Melville, NY, USA) and a field-emission scanning electron microscope (FE-SEM) (JEOL JSM-7610F, Akishima, Tokyo, Japan) were used for sample characterization. 

X-ray diffraction (XRD, Rigaku, Austin, TX, USA) was utilized in order to identify phases of AM Ti alloys and was carried out using a Rigaku D/MAX 2500/PC. The X-ray diffractometer was fitted with a CuKα radiation source. The specimens were scanned at ambient temperature at 2θ from 30° until 90° with a step size of 1° and a dwell time of 1 min per step. The XRD results were analyzed with the JADE9 (KS Analytical Systems, Aubrey, TX, USA) software.

### 2.3. Measurements of Electrochemical Critical Localized Corrosion Potential (E-CLCP) (ISO/CD 4631:2021)

An electrochemical polarization test cell was used with a Luggin capillary probe for the measurement of E-CLCP. An external reference electrode was used for measurement of the electrode potential, which was connected to the test cell. A carbon rod and a saturated calomel electrode (SCE) were used as a counter electrode and a reference electrode, respectively. The E-CLCP was measured as the repassivation potential by potentiodynamic–galvanostatic–potentiostatic processes. The test solution was the Ringer solution. Thirty-seven degrees Celsius was chosen as the temperature of the test solution, which is the human body temperature. The E-CLCP was determined in accordance with the following steps [30]:

(1) The test sample was formed into a rectangular sheet without a crevice former.
(2) Potentiodynamic anodic polarization was carried out from an open-circuit potential until an anodic current density rose to 500 μA/cm² by 1 mV/s potential-sweep velocity using a potentiostat.
(3) When the anodic current density reached 500 μA/cm², it was immediately held constant for 2 h.
(4) After holding the constant current density of 500 μA/cm² during 2 h, a constant polarization was immediately held in the reverse (cathodic) direction at an electrode potential of 10 mV, which was lower than the initial potential. As soon as the increase in current density was observed in the anodic direction, the constant potential was further decreased by another 10 mV. This operation was repeated until no further increase in the current density was found in the anodic direction after holding a constant potential for 2 h.
(5) The E-CLCP of AM Ti alloy specimens was determined at the highest potential value where no further increases in the current density were found in the anodic direction after holding a constant potential for 2 h.

The E-CLCP measurements were performed three times to ensure data reproducibility.

### 2.4. Measurements of Electrochemical Critical Localized Corrosion Temperature (ISO 22910:2020)

For the measurement of E-CLCT, an electrochemical polarization test cell containing a Luggin capillary probe was used and connected to an external reference electrode for measuring the electrode potential. An SCE and a carbon rod were used as the reference electrode and counter electrode, respectively. The E-CLCT was measured as the lowest temperature on the surface of the AM Ti alloy specimen at which stable localized corrosion occurred under specified test conditions (i.e., deaerated 25 wt% NaCl aqueous solution). The E-CLCT was measured according to the following steps [25,29]:

(1) The test sample was shaped into a rectangular sheet without a crevice former.
(2) The open-circuit potential of the test specimen was recorded during 1 h, and the desired anodic potential was applied to the specimen. The recommended applied potential for the Ti alloys (i.e., Ti–6Al–4V) in the concentrated 25 wt% NaCl aqueous solution was 2.8 V.
(3) If uncertainty existed concerning whether 2.8 V was sufficiently high to obtain the potential-independent E-CLCT, a test at 2.9 V was performed. If there was a significant deviation between the E-CLCT obtained at 2.8 and 2.9 V, there was a need for re-evaluation.
(4) Following the application of the potential for 60 s or longer, the temperature increased at a controlled rate.
(5) The current and solution temperatures were monitored throughout the test.
(6) E-CLCT was defined as the temperature at which a sharp increase in current density occurred during the temperature ramp at 1 °C/min.

E-CLCT measurements were performed three times to ensure data reproducibility.

### 2.5. Measurements of Electrochemical Impedance Spectroscopy and Mott–Schottky Plots Using Microdroplet Cells

**Figure 1:**
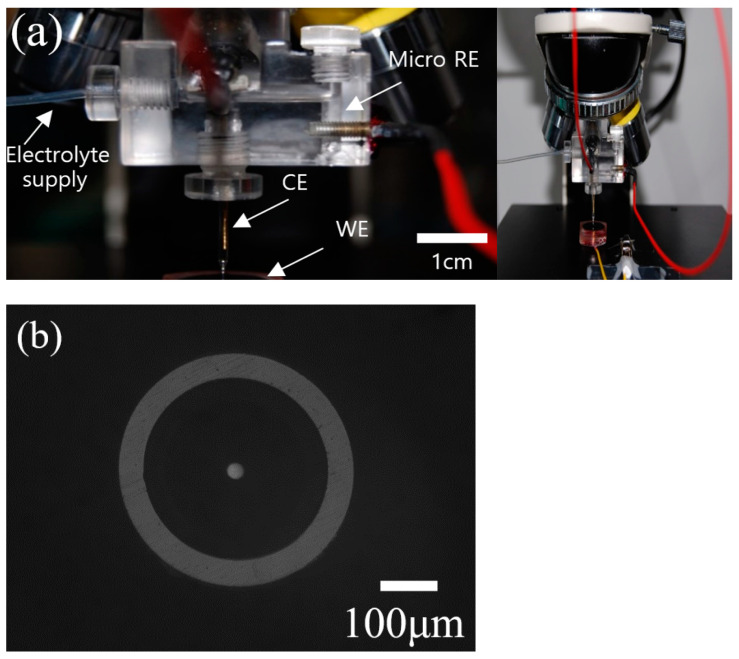
Experimental setup of (**a**) the specially designed microdroplet cell and (**b**) the diameter of the capillary tip (RE: reference electrode; WE: working electrode; CE: counter electrode).

The EIS measurements at the OCPs were carried out at a potentiostatic alternating current (AC) voltage amplitude of 10 mV within the frequency range of 10^−1^ to 10^3^ Hz after the E-CLCP and E-CLCT tests. The microdroplet cell setup for EIS test [21,22] is shown in Figure 1a. The microdroplet cell technique enabled the microelectrode to be aligned in a suitable location of the working electrode, and the direct measurement of local currents could be obtained during electrochemical polarization. A capillary tip with a diameter of 330 μm [21] Figure 1b was fabricated for the microdroplet cell tests. Silicon paste was used to seal the capillary in order to prevent a leakage of the solution from the capillary. The capillary was located in repassivated (E-CLCP) and corroded (E-CLCT) sites on the surface of the test samples. The EIS results were obtained using Gamry’s Echem Analyst software. 

The Mott–Schottky measurements were performed on the samples after testing occurred on the repassivated site (E-CLCP) and the corroded site (E-CLCT). The capacitance was measured at 1000 Hz using a potentiostat (Gamry PCIB-4750) via a microdroplet cell. Polarization was applied at successive steps of 50 mV, beginning in the cathodic direction at 1.0 V up to −2.0 V. EIS and Mott–Schottky plot measurements were performed three times to ensure data reproducibility in the Ringer solution.

### 2.6. Measurements of Repassivation Kinetics Using Abrading Electrode Tests

In order to obtain information on the repassivation kinetics of oxide films repassivated on the surface of the samples after exposure of their bare surfaces to the solution, an abrading electrode technique was chosen [43]. In order to obtain current transients, the electrode was abraded with a SiC disc while immersed in the electrolyte. The sample was mounted in the center of an epoxy resin with an exposed area of 1.8 × 10^−1^ cm^2^, which was welded with a copper wire, i.e., a current collector. The sample surface was abraded for renewing with a 2000-grit SiC disc. The disc was attached to the rotating shaft operated by a DC motor. An SCE was used as the reference electrode and was located closest to the working electrode. A carbon rod was selected as the counter electrode. The resulting current transient was acquired at a constant applied potential of 1.0 V. This is the potential region of the passive state of AM Ti–6Al–4V alloys [22] that begins a moment just after interrupting the abrading action on the specimen. Abrading tests were performed three times to ensure data reproducibility in the deaerated Ringer solution at 37 °C.

## 3. Results and Discussion

### 3.1. Studies of Microstructures

**Figure 2:**
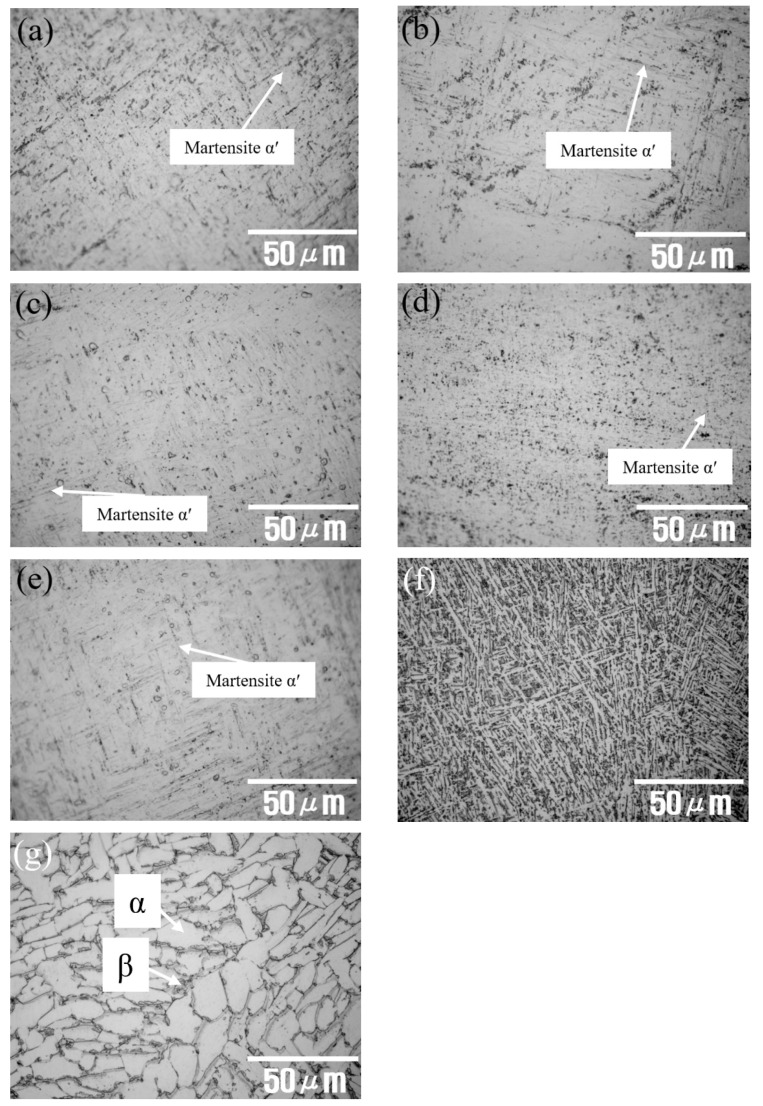
Optical micrographs of the AM Ti–6Al–4V alloys: (**a**) as-received sample and heat-treated samples: (**b**) 650HT, (**c**) 650WQ, (**d**) 750HT, (**e**) 750WQ, (**f**) 850HT, and (**g**) 1000HT (HT: furnace cooling; WQ: water quenching).

**Figure 3:**
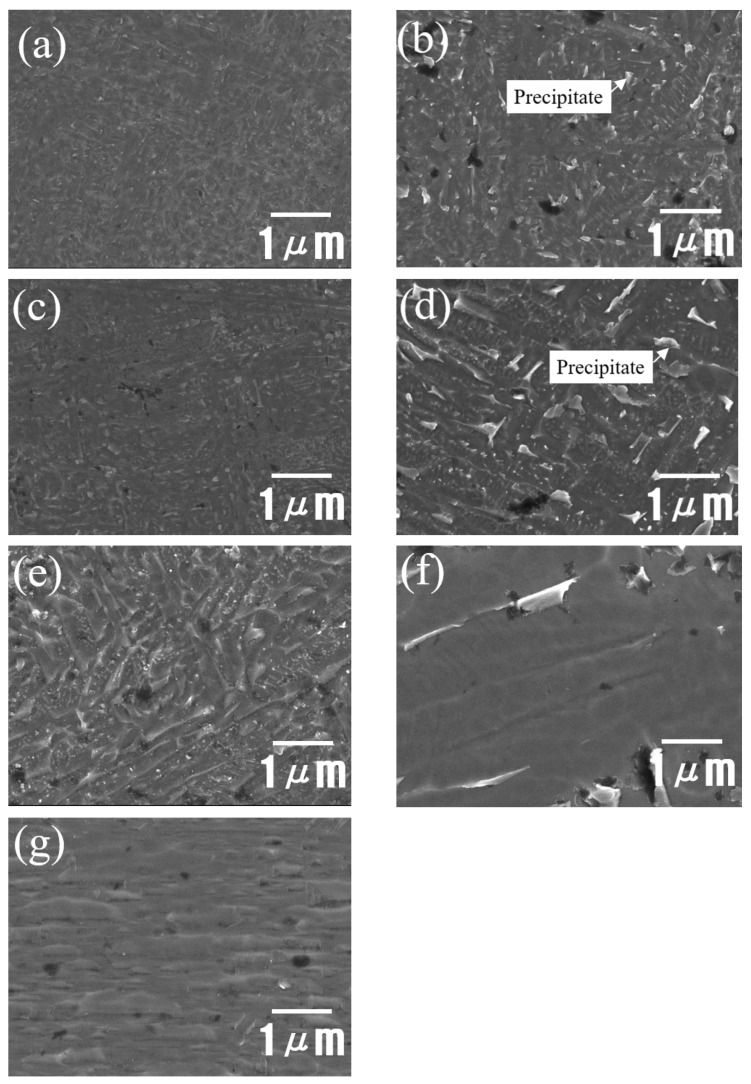
FE-SEM micrographs of the AM Ti–6Al–4V alloys: (**a**) as-received sample and heat-treated samples: (**b**) 650HT, (**c**) 650WQ, (**d**) 750HT, (**e**) 750WQ, (**f**) 850HT, and (**g**) 1000HT (HT: furnace cooling; WQ: water quenching).

As-received and HT AM Ti–6Al–4V alloys were observed by using an OM. Figure 2a shows the micrograph of an as-received AM Ti–6Al–4V alloy where the acicular martensite α′ phases are presented. Figure 2b–g show the micrographs of 650HT, 650WQ, 750HT, 750WQ, 850HT, and 1000HT AM Ti–6Al–4V alloys, respectively. All AM Ti–6Al–4V alloy specimens (650HT, 650WQ, 750HT, and 750WQ) show acicular martensite α′ phases because 650 °C and 750 °C are below the martensite-finishing temperature [44,45] (i.e., M_f_ ≈ 800 °C for Ti–6Al–4V). Therefore, pre-existing α′ phases still remain (without decomposition). The 850HT alloy shows a fine lamellar-like α phase [46,47], whereas the 1000HT alloy exhibits the mixed structure of lamellar α and β phases [48]. Figure 3a–e are the FE-SEM micrographs of the AM Ti–6Al–4V alloys for the as-received, 650HT, 650WQ, 750HT, and 750WQ samples, respectively. The 650HT and 750HT Ti–6Al–4V alloys contain nano-sized precipitates caused by aging due to the 600–700 °C heat treatment [41,49,50]. Although the precipitates may play some role in increasing mechanical strength, the precipitates (e.g., Ti_3_Al) are known to reduce the resistance to localized corrosion (e.g., pitting) [51,52,53]. Therefore, both the α′ phases and the precipitates formed in the 650HT and 750HT samples may be responsible for decreased resistance to localized corrosion [53,54]. However, the 650WQ and 750WQ samples contained fewer precipitates than the furnace cooled 650HT and 750HT samples did. This observation was already confirmed by Seo and Lee [41] by using microhardness tests that demonstrated that the values of the 650WQ and 750WQ samples were lower than those of the 650HT and 750HT samples.

Figure 3f,g are FE-SEM micrographs of the 850HT and 1000HT samples. They do not show any noticeable precipitates because precipitates are dissolved above 850 °C [55]. According to the XRD measurements performed by Seo and Lee [41], 1000HT is above the β-transus temperature (T_β_, approximately 995 °C). When a sample contains β phases (12.7% for volume fraction), it is known to increase corrosion resistance [14]. 

**Figure 4:**
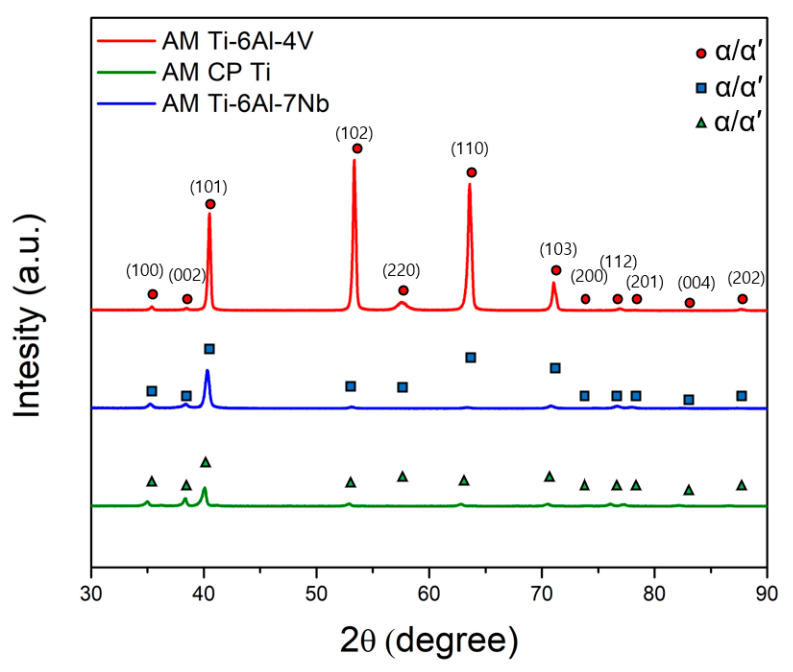
XRD patterns of the AM Ti–6Al–4V, AM Ti–6Al–7Nb, and AM CP Ti alloys.

The XRD patterns of the AM Ti–6Al–7Nb and AM CP Ti alloys were investigated in order to compare the AM Ti–6Al–4V alloy. As shown in Figure 4, the XRD measurements of the AM Ti–6Al–7Nb alloys were identified as an α phase with no β phase owing to rapid cooling during AM processing. XRD measurements cannot identify the difference between the α′ and α phases in the case of the same hexagonal closest packed structure [21]. The phase of AM CP Ti (single phase) was also identified as α. 

### 3.2. Determination of Electrochemical Critical Localized Corrosion Potential (E-CLCP) of Additive Manufactured Titanium Alloys

**Figure 5:**
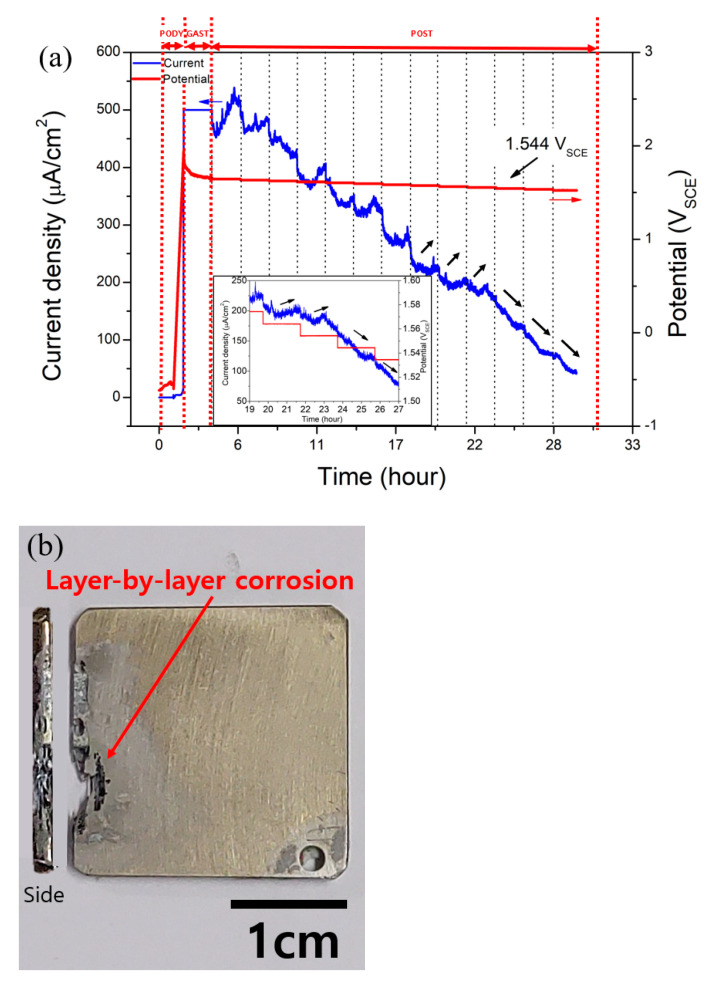
(**a**) Determination of E-CLCP values for the as-received AM Ti–6Al–4V alloy at 37 °C in Ringer solution (note that the small arrow indicates the direction of current flow); (**b**) the shape of the layer-by-layer corroded area of the as-received AM Ti–6Al–4V alloy. Inset is the magnified area around E-CLCP for the corresponding graph. (PODY: potentiodynamic; GAST: galvanostatic; POST: potentiostatic polarization).

**Figure 6:**
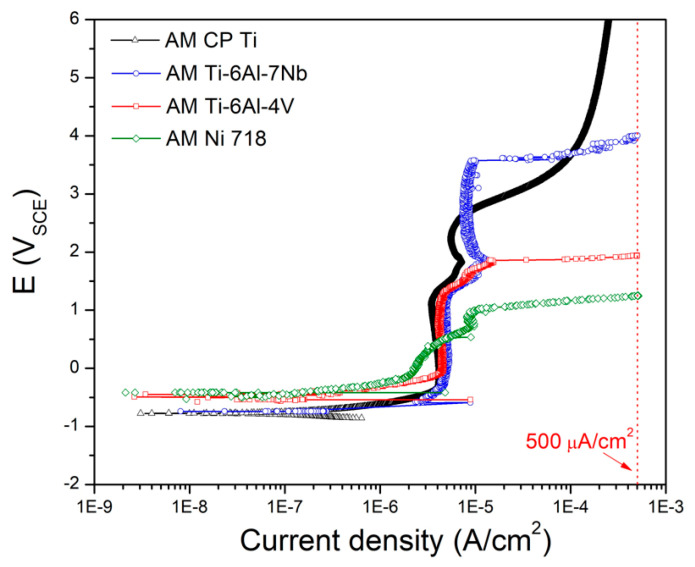
Potentiodynamic anodic curves of AM alloys such as CP Ti, Ti-6Al-7Nb, T-6Al-4V, and Ni 718. (Note 500 μA/cm^2^ is chosen as the suitable galvanostatic current density for galvanostatic mode.).

**Figure 7:**
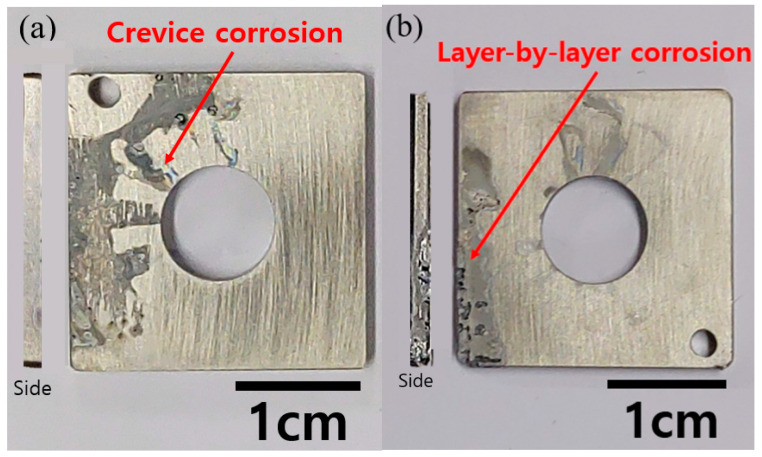
Different shapes of the corroded areas between (**a**) crevice corrosion of SM Ti-6Al-4V alloy and (**b**) layer-by-layer corrosion of AM Ti–6Al–4V alloy.

Figure 5a,b demonstrate the determination of the E-CLCP and the shape of a specimen after testing, respectively. The E-CLCP of the AM Ti–6Al–4V alloy samples was determined by the highest potential value where there was no further increase in current density in the anodic direction. The E-CLCP of AM Ti–6Al–4V was obtained as 1.544 (±0.002) V in the Ringer solution at 37 °C. As distinct from ASTM G192, any crevice assembly including a crevice former and a hole at the center of the specimen was not applied. In addition, in the beginning stage (i.e., at the galvanostatic mode), galvanostatic current density was applied up to 500 μA/cm^2^ (note: 2 μA/cm^2^ for Alloy 22 of ASTM G192 [36]). Based on the potentiodynamic anodic polarization curve of Figure 6, 500 μA/cm^2^ is chosen and applied as the suitable galvanostatic current density for galvanostatic mode causing localized corrosion such as the layer-by-layer corrosion characterized by AM Ti alloys. Figure 7a shows the crevice corrosion of SM Ti-6Al-4V underneath a crevice former while Figure 7b displays the layer-by-layer corrosion of AM Ti-6Al-4V alloys instead of the crevice corrosion under the crevice former. Layer-by-layer corrosion is initiated at the boundaries or edges of the AM Ti-6Al-4V specimen and proceeds to propagate. 

### 3.3. The Validity of Electrochemical Critical Localized Corrosion Potential in Comparison with Electrochemical Critical Localized Corrosion Temperature

**Figure 8:**
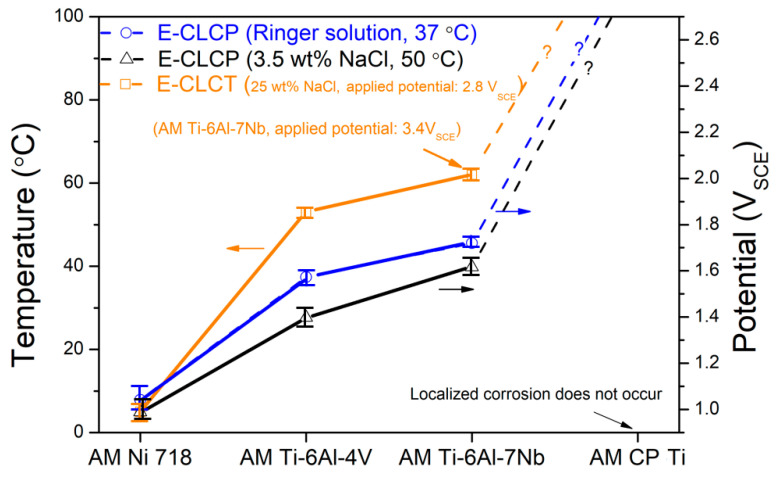
The comparison between the E-CLCT (ISO 22910:2020 [29]) and E-CLCP (ISO/CD 4631: 2021 [30]) values of the AM Ni 718 and AM Ti alloys (note that the E-CLCT test for the AM Ti–6Al–7Nb alloy was performed at the applied potential of 3.4 V_SCE_).

The demonstration of the validity of E-CLCP tests of AM Ti alloys was attempted in comparison with E-CLCT tests. The test method for E-CLCT is well described in ISO 22910:2020 [29]. In order to investigate the validity of E-CLCP tests of AM Ni718, AM Ti–6Al–4V, AM Ti–6Al–7Nb, and AM CP Ti alloys, the E-CLCT test results performed in concentrated NaCl (25 wt%) aqueous solution were compared with E-CLCP results. The E-CLCP tests were performed in NaCl (3.5 wt%) aqueous solution at 50 °C and in a Ringer solution at 37 °C. Figure 8 shows the correlation of the test results between E-CLCP and E-CLCT, representing the same tendency between E-CLCT and E-CLCP. This tendency in resistance against localized corrosion is as follows: AM CP Ti > AM Ti–6Al–7Nb > AM Ti–6Al–4V > AM Ni 718. It is obvious that the AM Ni 718 alloy demonstrates the lowest resistance to localized corrosion in comparison with the other AM Ti alloys. The potential 2.8 V was applied to the AM Ti–6Al–4V alloy for the E-CLCT test, whereas 3.4 V was applied to the AM Ti–6Al–7Nb alloy to maintain the potential-independent region on both alloys. A higher potential was applied to AM Ti–6Al–7Nb due to its higher localized corrosion resistance [25]. Therefore, based on the correlation between E-CLCP and E-CLCT showing the same tendency (as is shown in Figure 8), the newly proposed E-CLCP test method can be utilized to determine the resistance against localized corrosion on biomedical AM Ti alloys in human body environments. 

### 3.4. The Electrochemical Critical Localized Corrosion Potential and Electrochemical Critical Localized Corrosion Temperature of Additive Manufactured Ti–6Al–4V According to Various Heat Treatments in Terms of Localized Corrosion Resistance

**Figure 9:**
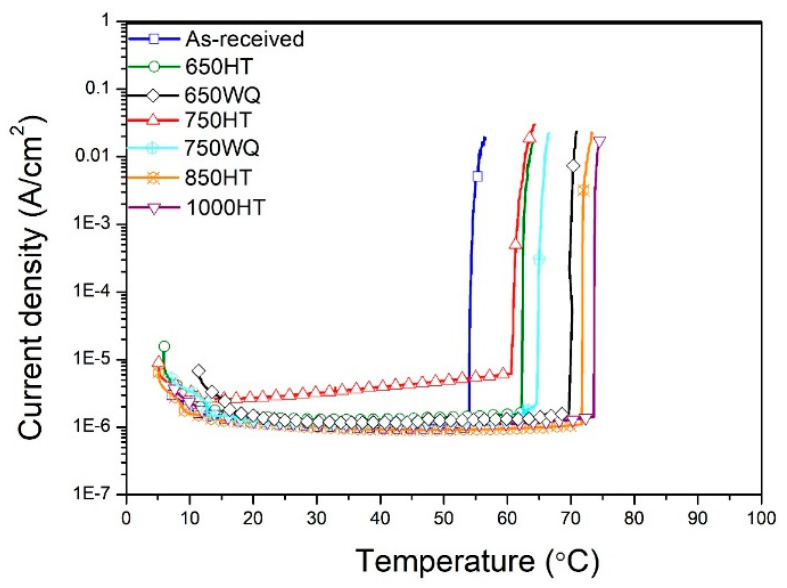
Determination of the E-CLCT values for the as-received and heat-treated AM Ti–6Al–4V alloys at various temperatures and cooling methods according to the applied potentials of 2.8 V_SCE_ in a concentrated NaCl (25 wt%) solution (HT: furnace cooling; WQ: water quenching).

**Figure 10:**
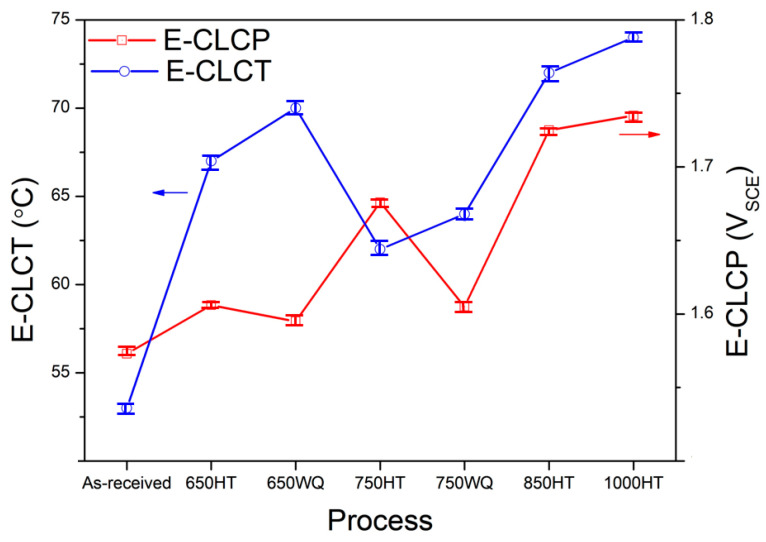
Comparison between E-CLCT and E-CLCP values for the as-received and heat-treated AM Ti–6Al–4V alloys (HT: furnace cooling; WQ: water quenching).

Although E-CLCP was measured under mild corrosive conditions (such as in the human body), the validity of evaluating localized corrosion resistance of the AM Ti alloys was demonstrated by comparison with E-CLCT. However, the mechanisms of resistance to localized corrosion on the as-received and HT AM Ti–6Al–4V alloys under E-CLCT and E-CLCP conditions differ at various temperatures because of the different localized corrosion and repassivation properties. E-CLCT is mainly measured for the initiation of localized corrosion on the AM Ti alloys based on temperature, whereas E-CLCP yields a repassivation potential of regenerated passive films of AM Ti alloys after breaking down. Figure 9 and Table 2 show the results of E-CLCT between the as-received and HT AM Ti–6Al–4V alloys in various HT temperatures. The values of localized corrosion resistance in the AM Ti–6Al–4V alloys measured by E-CLCT are sequenced as follows: 1000HT > 850HT > 650WQ > 650HT > 750WQ > 750HT > as-received. Due to decomposited martensite α′ phases in 850HT and 1000HT samples, their values of localized corrosion resistance are higher than the other samples. In addition, the 1000HT sample contains β phases, resulting in the best localized corrosion resistance among all samples [41,46]. The reduced localized corrosion resistance of the 650HT and 750HT samples is attributed to the pre-existing α′ phases and their precipitates. However, the 650WQ and 750WQ samples have better localized corrosion resistance than the 650HT and 750HT samples, demonstrating that water quenching prevents the formation of precipitates [41]. E-CLCP shows somewhat different results in Figure 10. The values of E-CLCP in the AM Ti–6Al–4V alloys are sequenced as follows: 1000HT > 850HT > 750HT > 750WQ ≈ 650HT > 650WQ > as-received. 

Table 2 shows that the E-CLCP value of the 750HT sample is higher than that of the 750WQ sample. The 650HT sample also has a higher E-CLCP value than the 650WQ sample. These results indicate that the precipitates have a beneficial effect on the repassivation of the passive film in terms of localized corrosion resistance. Buhl [56] also reported that precipitates on Ti–6Al–4V alloys would facilitate the repassivation process and maintained that repassivation occurred in accordance with a dissolution–precipitation mechanism. Based upon Buhl’s reported mechanism, the precipitates (e.g., Ti_3_Al in the 750HT and 650HT samples) may impose a higher potential on the matrix around them as the cathode. Once the preferential initiation of localized corrosion occurs at the anodic precipitates, the matrix around the precipitates may be repassivated with ease. Therefore, precipitates such as Ti_3_Al in the 750HT and 650HT samples may play advantageous roles on the repassivation of the matrix (compared with the 750WQ and 650WQ samples).

### 3.5. Studies on the Difference between Electrochemical Critical Localized Corrosion Potential and Electrochemical Critical Localized Corrosion Temperature in Localized Corrosion Resistance via Electrochemical Tests

EIS measurements using microdroplet cells

**Figure 11:**
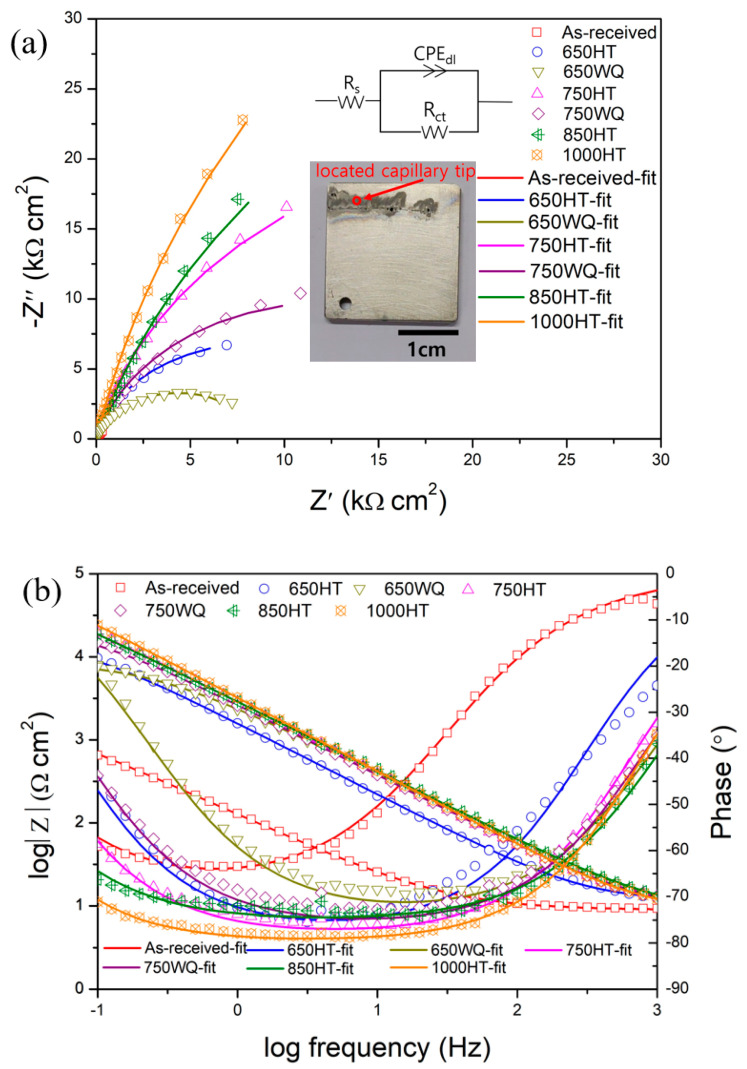
(**a**) Nyquist and (**b**) Bode plots of EIS for the corroded samples after E-CLCP for the as-received, HT, and WQ samples of AM Ti–6Al–4V alloys in Ringer solution. The insets show the equivalent circuit for fitting and the optical micrograph with the location of capillary tip in a microdroplet cell, respectively (HT: furnace cooling; WQ: water quenching).

**Figure 12:**
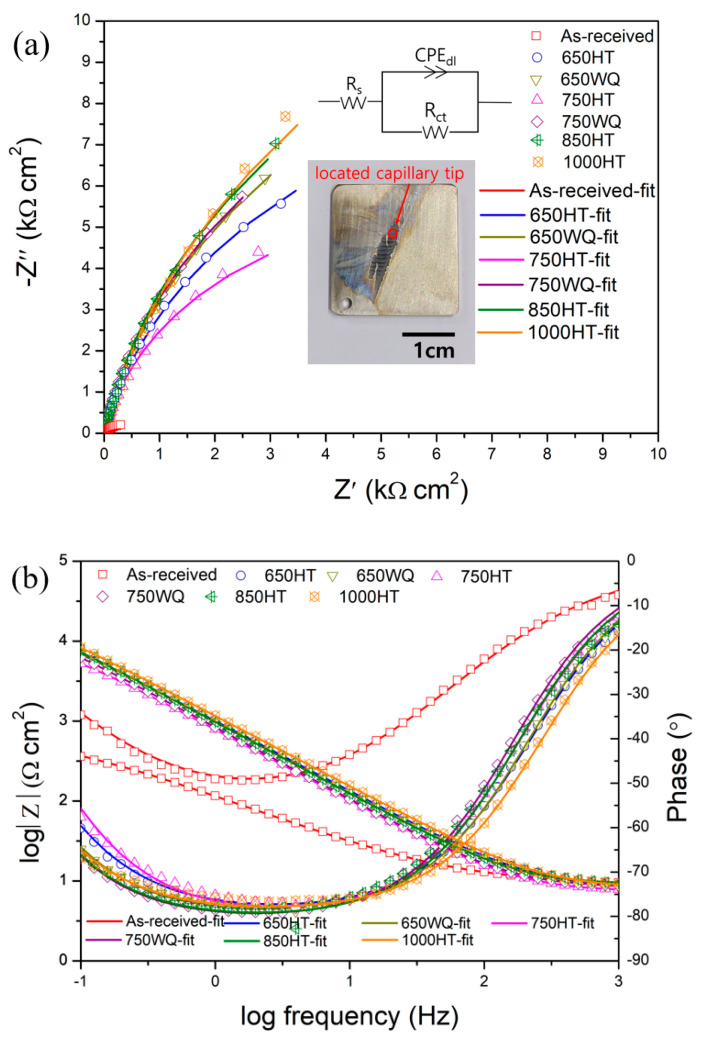
(**a**) Nyquist and (**b**) Bode plots of EIS for the corroded samples after E-CLCT for the as-received, HT, and WQ samples of AM Ti–6Al–4V alloys in Ringer solution. The insets show the equivalent circuit for fitting and the optical micrograph with the location of capillary tip in microdroplet cell, respectively (HT: furnace cooling; WQ: water quenching).

EIS measurements were conducted using microdroplet cells. Figure 11 and Figure 12 (a: Nyquist plot; b: Bode plot) and Table 3 and Table 4 show the EIS measurement results on repassivated or corroded sites in samples after E-CLCP and E-CLCT tests, respectively. According to the Bode plots (after the E-CLCP tests) shown in Figure 11b, the phase angles are flat in the middle-frequency (1–10 Hz) region. A plateau close to −90° corresponds to a dense oxide film [57] and shows the following sequence in the AM Ti–6Al–4V alloys: (≈−80°) 1000HT > 850HT > 750HT > 750WQ > 650HT > 650WQ > as-received (≈−65°). The 650HT or 750HT samples with precipitates are the more protective passive films compared with the 650WQ or 750WQ samples without precipitates, respectively. The higher plateaus of the as-received, 650WQ, and 750WQ samples (than the plateaus of the 650HT and 750HT samples) indicate that they are the less stable passive films after repassivation. Figure 12 shows the Nyquist (a) and Bode (b) plots of the samples after the E-CLCT tests. As is shown in the Bode plot, after the E-CLCT tests, the plateaus of the phase angles are in the middle-frequency (1–10 Hz) region. The dense oxide film in the AM Ti–6Al–4V alloys is sequenced as follows: (≈−80°) 1000HT > 850HT > 650WQ > 650HT > 750WQ > 750HT > as-received (≈−50°). Based upon these test results, precipitates in the 650HT and 750HT samples may play an important role on repassivation in mild corrosive environments (such as simulated human body conditions, E-CLCP). In harsh environments (such as 25% NaCl aqueous solution, and E-CLCT), precipitates may provide the initiation site for localized corrosion instead of repassivation.

The Randles circuit model was used to examine an equivalent circuit consisting of a solution resistance (*R_s_*), a charge transfer resistance (*R_ct_*), and constant phase elements (*CPE_dl_*). An electrochemical reaction from the ideal capacitor behavior is deviated due to the effects of surface inhomogeneity and roughness described by CPE. The estimated interfacial capacitance is defined in Equation (1) [58]:

(1) $$C_{eff}={\left[Q{\left\{\frac{R_{s}+R_{ct}}{R_{s}R_{ct}}\right\}}^{\left(\alpha -1\right)}\right]}^{\frac{1}{\alpha }}$$

where *Q* is the CPE coefficient value, and *α* is the exponent of CPE ranging from −1 to 1 (related to non-uniform current distribution). 

Accordingly, *α* in Table 3 represents the deviation from the ideal capacitor, which is defined by *α* = 1. The capacitance should be related to film thickness *d_eff_* according to Equation (2):

(2) $$d_{eff}=\frac{{\varepsilon \varepsilon }_{0}}{C_{eff}}$$

where *ε* is the dielectric constant of a passive film (considered to be 85) [59,60], and *ε_o_* is the vacuum permittivity of the free space (8.854 × 10^−14^ F cm^−1^). 

**Figure 13:**
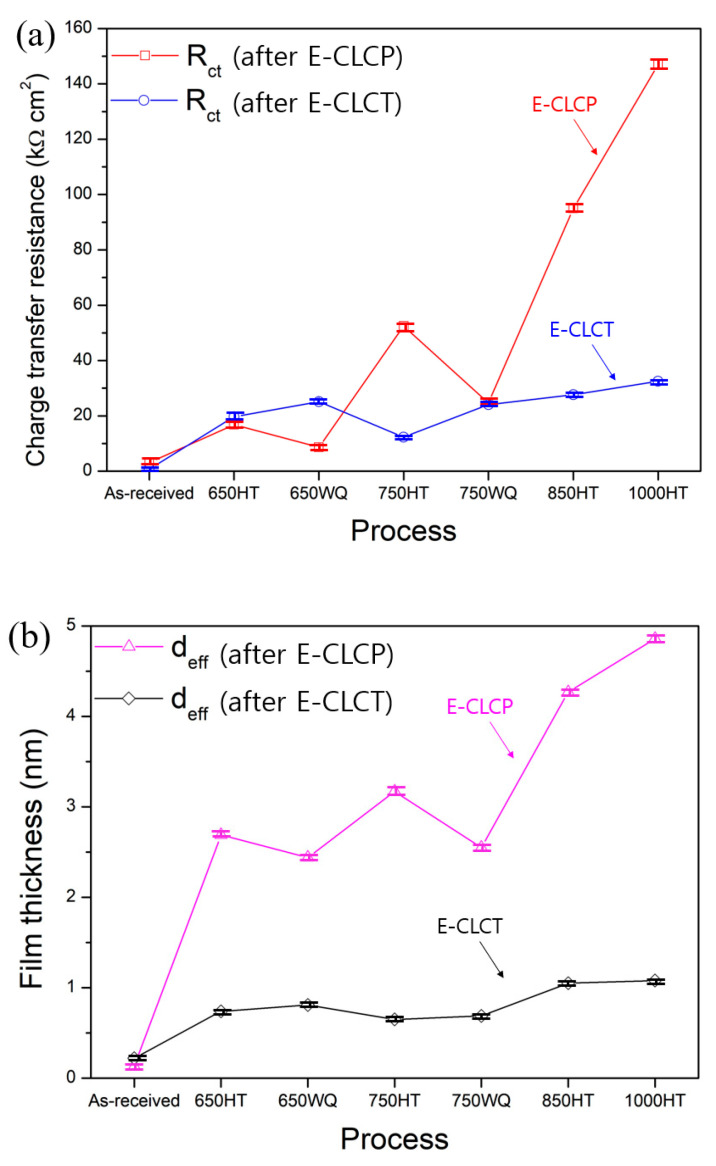
Comparison of (**a**) *R_ct_* of EIS tests and (**b**) thickness of passive films (*d_eff_*) between the E-CLCP and E-CLCT tests for the as-received and various heat-treated AM Ti–6Al–4V alloys using furnace cooling or water quenching methods (HT: furnace cooling; WQ: water quenching).

The values of *C_eff_* and *d_eff_* are presented in Table 3 and Table 4. Figure 13a shows the *R_ct_* values of all specimens measured after E-CLCP and E-CLCT tests. The results demonstrate that the *R_ct_* values of the 650HT or 750HT samples are higher than those of the 650WQ or 750WQ samples after the E-CLCP tests. The results also demonstrate that the *R_ct_* values of the 650HT and 750HT samples are lower than those of the 650WQ and 750WQ samples after the E-CLCT tests. The 850HT and 1000HT samples show higher *R_ct_* values in both E-CLCT and E-CLCP compared with every other sample due to the complete decomposition of α′ phases and the emergence of β phases. Furthermore, for the calculated *d_eff_* values, Figure 13b represent the same tendency of the *R_ct_* values obtained after E-CLCP and E-CLCT tests values. This indicates that the repassivation films formed in the E-CLCP tests are thicker than the passive films formed in the E-CLCT tests. 

Mott–Schottky analysis using microdroplet cells

**Figure 14:**
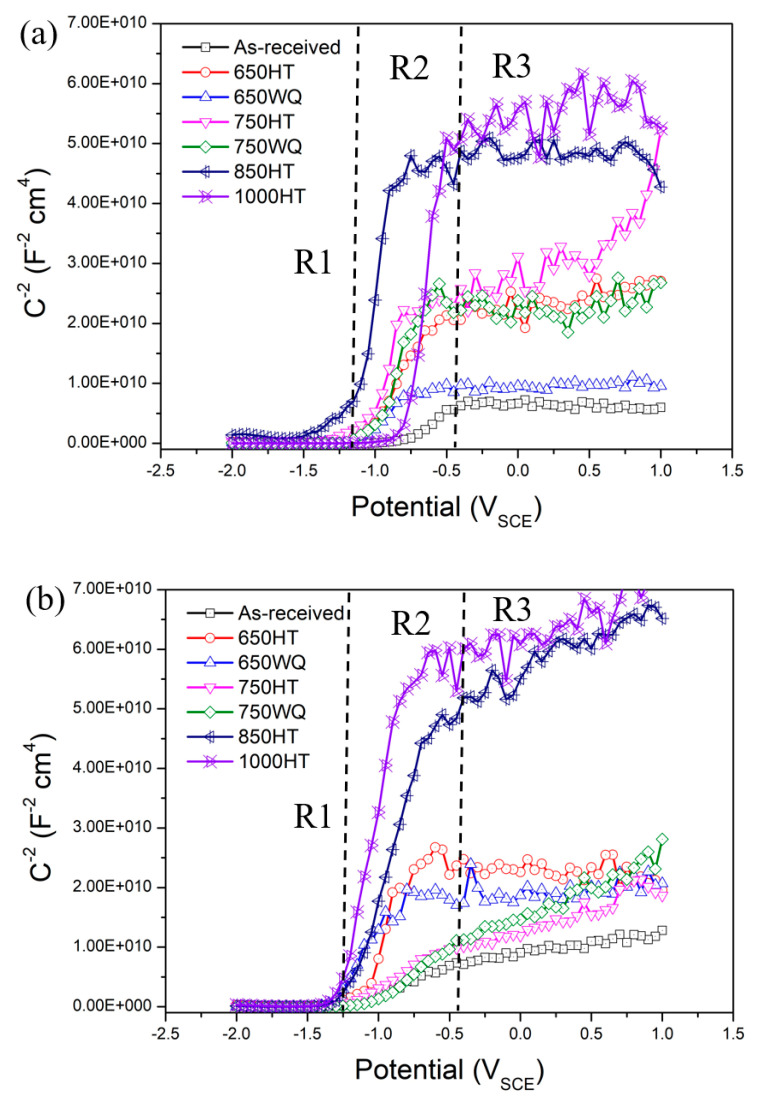
Mott–Schottky plots for the corroded samples after (**a**) E-CLCP and (**b**) E-CLCT for the as-received, HT, and WQ samples of the AM Ti–6Al–4V alloys in Ringer solutions (HT: furnace cooling; WQ: water quenching).

In order to further understand the properties of passive films in the samples after E-CLCP and E-CLCT tests, a Mott–Schottky analysis was performed separately on each passive film using a microdroplet cell. Figure 14a,b show the Mott–Schottky plots (1/C^2^ versus E) after E-CLCP and E-CLCT tests. The capacitance measurement results reveal the existence of three potential ranges (R1–R3) due to different capacitance behaviors with applied potentials. The AM Ti–6Al–4V alloys exhibit an n-type semiconductor of the Ti oxide layer, as is shown by the positive slopes in the R2 range (Figure 14). 

The different donor densities in the R2 range of the individual samples of the AM Ti–6Al–4V alloys for n-type semiconductors can be calculated using Equation (3) [60,61,62,63,64]:

(3) $$\frac{1}{C_{sc}^{2}}=\frac{2}{\varepsilon \varepsilon_{0}eN_{D}}\left(E-E_{fb}-\frac{kT}{e}\right)$$

where *ε* is the dielectric constant of a passive film (considered to be 85) [60], *ε*_0_ is the vacuum permittivity of the free space (8.854 × 10^−14^ F cm^−1^), *e* is the electron charge (1.602 × 10^−19^ C), *N_D_* is the donor density, *E_fb_* is the flat band potential, *k* is the Boltzmann constant, and *T* is the absolute temperature. The term *N_D_* can be determined on the basis of the slopes of the Mott–Schottky plots.

The calculated donor densities are approximately 10^18^–10^20^ cm^−3^ and are summarized in Table 5. The sequence of the donor densities on the repassivated regions of the AM Ti–6Al–4V alloys after the E-CLCP tests is as follows: 1000HT > 850HT > 750HT > 750WQ > 650HT > 650WQ > as-received. The sequence of the donor densities of the corroded regions of the AM Ti–6Al–4V alloys after the E-CLCT tests is as follows: 1000HT > 850HT > 650WQ > 650HT > 750WQ > 750HT > as-received. 

**Figure 15:**
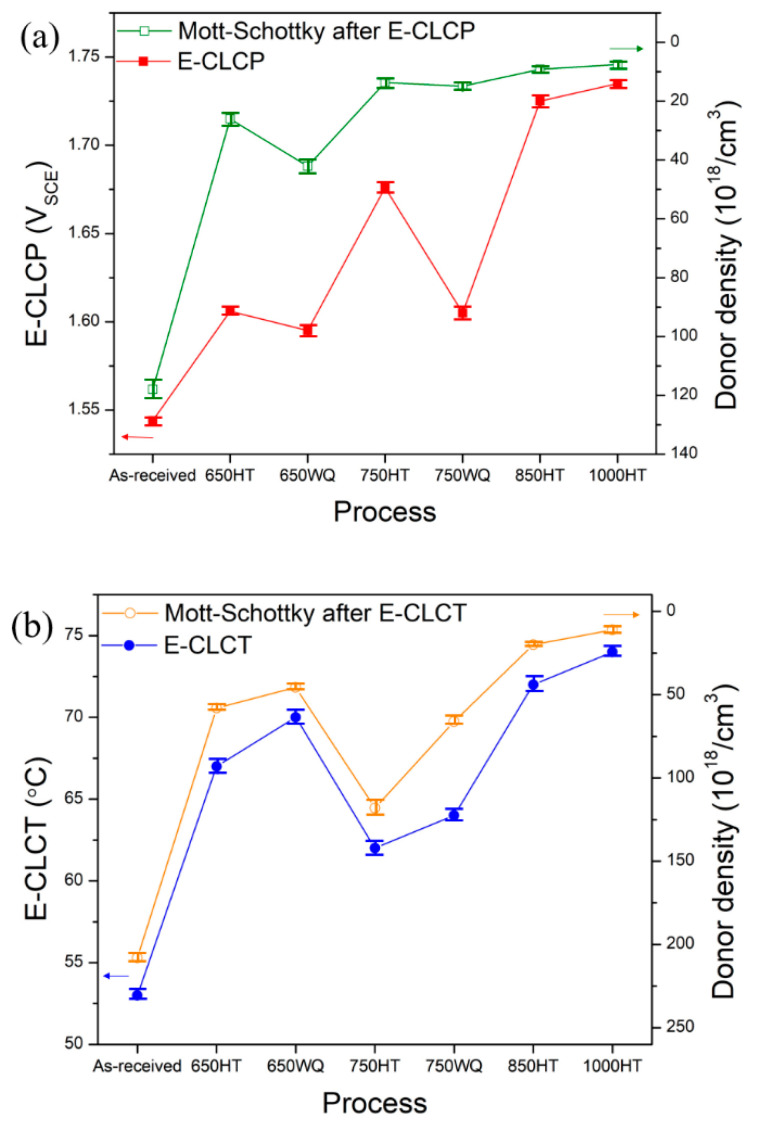
Comparison between (**a**) donor densities and E-CLCP and (**b**) donor densities and E-CLCT for the as-received and heat-treated AM Ti–6Al–4V alloys using furnace cooling or water quenching methods (HT: furnace cooling; WQ: water quenching).

These sequences agree exactly with the sequence of localized corrosion resistance obtained by E-CLCP or E-CLCT shown in Figure 15a,b, respectively. The point defect model (PDM) [65,66,67,68] enabled understanding the relationship between donor density and the localized corrosion resistance measured by E-CLCP or E-CLCT. According to PDM, the passive film contains several point defects of oxygen or cation vacancies that serve as donors or acceptors, respectively. Therefore, an increased donor density in the passive film may be regarded as an increase in oxygen vacancies. Roh and Macdonald [66,67] reported that oxygen vacancy dominates over the metal interstitials in determining the defect structure of the n-type passive film of Ti alloys. Jiang et al. [68] investigated the pitting resistance of Ti based on a modified PDM. Therefore, the susceptibility of the localized corrosion resistance to the repassivated or corroded regions after the E-CLCP and E-CLCT tests is attributed to the donor densities of the as-received and HT AM Ti–6Al–4V alloys. The higher the donor density, the more susceptible the sample is to localized corrosion. 

Repassivation kinetics using abrading electrode tests

**Figure 16:**
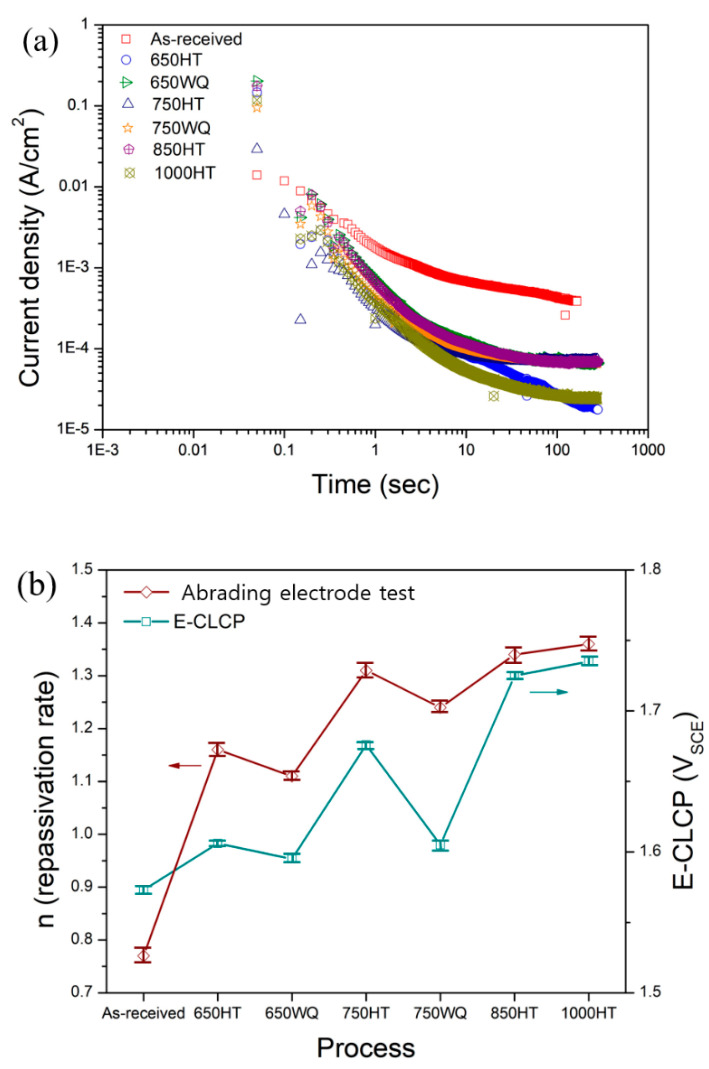
Plots of *i*(*t*) vs. time in logarithmic scale for (**a**) all AM Ti–6Al–4V alloys and (**b**) the comparison between the repassivation rate obtained from the abrading electrode technique and E-CLCP (HT: furnace cooling; WQ: water quenching).

In order to investigate the difference between E-CLCP and E-CLCT in localized corrosion resistance, the repassivation kinetics were studied by using the abrading electrode technique. Figure 16a demonstrates the decay of current density with time in logarithmic scale during the repassivation of all HT AM Ti–6Al–4V samples. Figure 16b represents the relationship between the abrading electrode and the E-CLCP test results. The repassivation kinetics of the as-received and HT AM Ti–6Al–4V alloys can be expressed using Equation (4) [43]:

(4) $$i=At^{-n}$$

where  i designates anodic current density, A is the constant, t is the time, and n is the repassivation rate parameter indicating the slope of the logi−logt plot. Based on Equation (4), the value of n is regarded as an indirect measurement of the growth of the oxide film on a bare surface as a function of applied anodic potential. 

Table 6 exhibits the repassivation rates of the as-received and HT AM Ti–6Al–4V alloys. Figure 16b shows the comparison between the repassivation rates obtained from the abrading electrode and E-CLCP tests, indicating the same tendency between the repassivation rate and E-CLCP. The repassivation rate of 1.16 (±0.02) of the 650HT sample with precipitates is higher than the rate of 1.11 (±0.01) of the 650WQ sample without precipitates. The repassivation rate of 1.31 (±0.03) of the 750HT sample with precipitates is also higher than the rate of 1.24 (±0.02) of the 750WQ sample. These results indicate that precipitates may play an important role in repassivation kinetics by facilitating the repassivation rate. 

## 4. Conclusions

This study proposed a new method (E-CLCP) to evaluate the localized corrosion resistance of AM Ti alloys in biomedical solution environments. The procedures for determining E-CLCP are completely different from the E-CLCT and conventional CCP procedures. At the normal human body ranges of pH (7.4) and temperature (37 °C), the use of the E-CLCT measurement method should be limited because of the temperature scan required during testing. The test results show that the values of E-CLCT on AM Ti–6Al–4V alloys display 1000HT > 850HT > 650WQ > 650HT > 750WQ > 750HT > as-received while those of E-CLCP exhibit 1000HT > 850HT > 750HT > 750WQ ≈ 650HT > 650WQ > as-received in sequence. The difference between two independent test methods is because E-CLCT is primarily for the initiation of localized corrosion on AM Ti alloys based on temperature, whereas E-CLCP is measured as it yields the repassivation potential of the regenerated passive films of the AM Ti alloys after breaking down. E-CLCP displays localized corrosion resistance based upon repassivation kinetics in environments similar to the human body, whereas E-CLCT reflects localized corrosion resistance due to passive film breakdown in much harsher corrosive environments (e.g., 25 wt% NaCl aqueous solutions). Therefore, the newly proposed E-CLCP test must be the most suitable method to evaluate the resistance to localized corrosion on biomedical AM Ti alloys. 

## Figures and Tables

**Table 1:** Material composition of the AM alloys (wt%).

	C	Fe	Al	V	Sn	Nb	O	Mn	Si	Co	Mb	Cb	Cr	Ni	Ti
AM Ti-6Al-4V	0.011	0.18	6.1	3.7	0.01	-	-	-	-	-	-	-	-	-	Bal.
AM Ti-6Al-7Nb	0.01	0.125	6.05	-	-	7.1	0.1	-	-	-	-	-	-	-	Bal.
AM CP Ti	0.1	0.3	-	-	-	-	-	-	-	-	-	-	-	-	Bal.
AM Ni718	0.08	17.0	0.8	-	-	-	-	0.35	0.35	1.0	3.0	5.0	19.0	Bal.	0.6

AM: additive manufacturing.

**Table 2:** Values of E-CLCT and E-CLCP of the as-received and heat-treated AM Ti–6Al–4V alloys in various heat-treated temperatures.

	E-CLCT (°C)	E-CLCP (V_SCE_)
As-received	53 (±0.3)	1.544 (±0.002)
650HT	67 (±0.2)	1.606 (±0.004)
650WQ	70 (±0.4)	1.595 (±0.002)
750HT	62 (±0.2)	1.676 (±0.002)
750WQ	64 (±0.2)	1.605 (±0.002)
850HT	72 (±0.3)	1.725 (±0.003)
1000HT	74 (±0.4)	1.735 (±0.001)

HT: furnace cooling; WQ: water quenching.

**Table 3:** Fitting parameters of the EIS measured in Ringer solutions for the as-received and heat-treated AM Ti–6Al–4V alloys after E-CLCP tests.

	R_S_ (Ω cm^2^)	R_ct_ (kΩ cm^2^)	α	CPE_dl_, Q (Ω^−1^∙cm^−2^∙Sec^α^)	C_eff_ (μF cm^−2^)	d_eff_ (nm)
As-received	8.91 (±0.17)	3.10 (±0.27)	0.7727	1921 (±21) × 10^−6^	580 (±11)	0.13 (±0.02)
650HT	11.39 (±0.14)	16.8 (±0.42)	0.8653	82.8 (±5.2) × 10^−6^	27.9 (±0.9)	2.69 (±0.05)
650WQ	8.67 (±0.11)	8.65 (±0.18)	0.8290	126 (±8) × 10^−6^	30.9 (±1.2)	2.44 (±0.04)
750HT	9.99 (±0.46)	52.5 (±1.1)	0.8716	69.2 (±1.7) × 10^−6^	23.7 (±1.1)	3.17 (±0.15)
750WQ	8.76 (±0.24)	24.6 (±0.4)	0.8831	77.4 (±1.4) × 10^−6^	29.5 (±5.0)	2.55 (±0.07)
850HT	8.30 (±0.19)	95.0 (±2.7)	0.8380	73.5 (±1.2) × 10^−6^	17.6 (±0.8)	4.27 (±0.07)
1000HT	8.78 (±0.28)	147 (±9)	0.8473	60.5 (±1.4) × 10^−6^	15.5 (±0.7)	4.86 (±0.05)

HT: furnace cooling; WQ: water quenching.

**Table 4:** Fitting parameters of the EIS measured in Ringer solutions for the as-received and heat-treated AM Ti–6Al–4V alloys after the E-CLCT tests.

	R_S_ (Ω cm^2^)	R_ct_ (kΩ cm^2^)	α	CPE_dl_, Q (Ω^−1^∙cm^−2^∙Sec ^α^)	C_eff_ (μF cm^−2^)	d_eff_ (nm)
As-received	8.90 (±0.10)	0.76 (±0.03)	0.6547	2512 (±37) × 10^−6^	336 (±9)	0.22 (±0.01)
650HT	8.47 (±0.10)	19.7 (±0.5)	0.8839	232 (±16) × 10^−6^	102 (±1)	0.74 (±0.01)
650WQ	8.09 (±0.09)	25.1 (±1.5)	0.8919	201 (±17) × 10^−6^	92.3 (±0.8)	0.81 (±0.03)
750HT	7.44 (±0.08)	12.3 (±0.5)	0.8954	243 (±20) × 10^−6^	116 (±1)	0.65 (±0.08)
750WQ	8.42 (±0.07)	24.1 (±1.2)	0.8950	226 (±23) × 10^−6^	108 (±1)	0.69 (±0.07)
850HT	9.11 (±0.09)	27.7 (±1.9)	0.8646	193 (±17) × 10^−6^	71.4 (±0.5)	1.05 (±0.05)
1000HT	8.22 (±0.10)	32.5 (±2.2)	0.8836	168 (±14) × 10^−6^	70.5 (±0.4)	1.08 (±0.04)

HT: furnace cooling; WQ: water quenching.

**Table 5:** Mott–Schottky data for corroded sites after the E-CLCP and E-CLCT tests of the as-received and heat-treated samples of the AM Ti–6Al–4V alloys.

	Donor Density (×10^18^/cm^3^) after E-CLCP	Donor Density (×10^18^/cm^3^) after E-CLCT
As-received	118 (±5)	208 (±4)
650HT	26.0 (±1.9)	58.1 (±0.2)
650WQ	42.1 (±1.3)	45.6 (±1.5)
750HT	13.7 (±0.7)	118 (±9)
750WQ	14.9 (±0.6)	66.2 (±2.7)
850HT	9.16 (±0.8)	20.0 (±0.7)
1000HT	7.54 (±0.4)	11.2 (±0.8)

HT: furnace cooling; WQ: water quenching.

**Table 6:** Values of the repassivation rate n, obtained from the linear region slope of log *i*(*t*) vs. log *t* representation, for the as-received and heat-treated AM Ti alloys.

	n (Repassivation Rate)
As-received	0.77 (±0.03)
650HT	1.16 (±0.02)
650WQ	1.11 (±0.01)
750HT	1.31 (±0.03)
750WQ	1.24 (±0.02)
850HT	1.34 (±0.03)
1000HT	1.36 (±0.03)

HT: furnace cooling; WQ: water quenching.

## Data Availability

Data sharing not applicable.
